# Whole-Genome Analysis of Escherichia Phage vB_EcoM-S1P5QW, Isolated from Manures Collected from Cattle Farms in Maine

**DOI:** 10.1128/mra.00041-22

**Published:** 2022-03-07

**Authors:** Irwin A. Quintela, Anya Hwang, Tyler Vasse, Alexandra Salvador, Yujie Zhang, Yen-Te Liao, Vivian C. H. Wu

**Affiliations:** a Produce Safety and Microbiology Research Unit, U.S. Department of Agriculture, Agricultural Research Service, Western Regional Research Center, Albany, California, USA; Portland State University

## Abstract

Here, we report a complete genome sequence of Escherichia phage vB_EcoM-S1P5QW, a T4-like bacteriophage that was isolated from manures collected from cattle farms in Maine. Escherichia phage vB_EcoM-S1P5QW can infect Escherichia coli O26:H11 strains and is devoid of virulence, antibiotic resistance, and lysogeny-associated genes, which may be meaningful for further biocontrol studies.

## ANNOUNCEMENT

Shiga toxin-producing Escherichia coli (STEC) O26:H11 (STEC O26) has contributed to recent outbreaks associated with prepacked sandwiches in the United Kingdom and raw flour in the United States (1, 2). Novel strategies are needed to reduce or eliminate these biological hazards effectively. Lytic bacteriophages provide alternative solutions to the traditional control methods in food production (3). Here, we report a complete genome sequence of Escherichia phage vB_EcoM-S1P5QW (S1P5QW), a T4-like bacteriophage that was isolated from manure samples and can infect and lyse STEC O26:H11, as shown by plaque formation and the presence of lysis-protein-encoding genes based on whole-genome analysis. In brief, S1P5QW was isolated by enrichment (overnight at 37°C) of manure samples from an anonymous farm in central Maine with STEC O26:H11 strains in tryptic soy broth with 10% CaCl_2_ and was purified and concentrated using Amicon filters (Millipore, MA, US) and CsCl density gradient centrifugation, respectively (4). A phage DNA isolation kit (Norgen, Canada) was used to extract DNA. The TruSeq Nano DNA library preparation kit (Illumina, San Diego, CA) was used to construct a DNA library (2 × 250 bp) from 200 ng DNA before sequencing on an Illumina MiSeq system. The raw sequence reads (4,126,179 paired-end reads) were quality filtered and trimmed using FastQC (Galaxy v0.72) (5) and Trimmomatic (Galaxy v0.36.6) (6) at a Q30 threshold. *De novo* assembly was performed with the remaining quality reads (minimum contig length, >10,000 bp) using SPAdes v3.13.0 on the KBase server (7, 8). The resulting largest contig was identified as a phage genome by BLASTn and was annotated using both Prokka v1.14.5 (9) and RAST server (10) pipelines. Genome annotations were compared and curated using UniProt (11), BLASTp (12), and open reading frame (ORF) Finder (Geneious v11.0.4). PhageTerm (13) was used to determine the packaging mechanisms and genome termini, while the tRNAscan-SE v2.0 web server facilitated the prediction of tRNAs (14). The presence of virulence and antibiotic resistance genes was determined by VirulenceFinder v2.0 (15) and ResFinder v3.0 (16), respectively. All tools were run with default parameters unless otherwise specified.

S1P5QW, with morphology belonging to the *Myoviridae* family, has linear double-stranded DNA with a genome size of 166,102 bp, encoding 10 tRNAs, and an average G+C content of 35.5%. Based on BLASTn analysis (12), the phage shares 96.8% average nucleotide identity (96% query coverage) with *Shigella* phage SHBML-50-1 (GenBank accession number KX130864.1). The genomic features of S1P5QW are highly similar to those of T4 or T4-like phages. A total of 256 ORFs were found; 127 of those were annotated as encoding functional proteins, including 39 ORFs encoding DNA replication and regulation proteins, DNA helicase, DNA polymerase, clamp, reductase, and DNA primase. PhageTerm indicated that the S1P5QW phage genome was circularly permuted with random termini, but it was unable to determine the packaging mechanism. An inner membrane subunit of spanin shares 91.5% amino acid identity with its counterpart in phage T4 (GenBank accession number NP_049837). Lysis proteins (657-bp holin and 495-bp endolysin) confirmed the lytic activity of S1P5QW against STEC O26:H11. S1P5QW does not possess *stx*, lysogeny-associated, and antibiotic resistance genes, which is meaningful for STEC O26 biocontrol applications.

### Data availability.

The complete genome sequence of Escherichia phage vB_EcoM-S1P5QW has been deposited in GenBank under the accession number OL956808. The sequencing raw reads have been deposited under the BioSample accession number PRJNA790824. The version of the phage genome described in this paper is the first version.
